# Resolution and quantification of arginine, monomethylarginine, asymmetric dimethylarginine, and symmetric dimethylarginine in plasma using HPLC with internal calibration

**DOI:** 10.1002/bmc.3548

**Published:** 2015-07-30

**Authors:** Matthew S. Alkaitis, Glenn Nardone, Jessica H. Chertow, Hans C. Ackerman

**Affiliations:** ^1^ Laboratory of Malaria and Vector ResearchNational Institute of Allergy and Infectious DiseasesRockvilleMarylandUSA; ^2^ Radcliffe Department of MedicineUniversity of Oxford, John Radcliffe HospitalHeadingtonOxfordUK; ^3^ Research Technologies BranchNational Institute of Allergy and Infectious DiseasesRockvilleMarylandUSA

**Keywords:** ADMA, SDMA, L-NMMA, Arginine, Nitric Oxide Synthase, Vascular Homeostasis

## Abstract

*N^G^*,*N^G^*‐dimethyl‐l‐arginine (asymmetric dimethylarginine, ADMA),*N^G^*‐monomethyl‐l‐arginine (l‐NMMA) and *N^G^*,*N* ^*G*’^‐dimethyl‐l‐arginine (symmetric dimethylarginine, SDMA) are released during hydrolysis of proteins containing methylated arginine residues. ADMA and l‐NMMA inhibit nitric oxide synthase by competing with l‐arginine substrate. All three methylarginine derivatives also inhibit arginine transport. To enable investigation of methylarginines in diseases involving impaired nitric oxide synthesis, we developed a high‐performance liquid chromatography (HPLC) assay to simultaneously quantify arginine, ADMA, l‐NMMA and SDMA. Our assay requires 12 μL of plasma and is ideal for applications where sample availability is limited. We extracted arginine and methylarginines with mixed‐mode cation‐exchange columns, using synthetic monoethyl‐l‐arginine as an internal standard. Metabolites were derivatized with ortho‐phthaldialdeyhde and 3‐mercaptopropionic acid, separated by reverse‐phase HPLC and quantified with fluorescence detection. Standard curve linearity was ≥0.9995 for all metabolites. Inter‐day coefficient of variation (CV) values were ≤5% for arginine, ADMA and SDMA in human plasma and for arginine and ADMA in mouse plasma. The CV value for l‐NMMA was higher in human (10.4%) and mouse (15.8%) plasma because concentrations were substantially lower than ADMA and SDMA. This assay provides unique advantages of small sample volume requirements, excellent separation of target metabolites from contaminants and validation for both human and mouse plasma samples. © 2015 The Authors Biomedical Chromatography published by John Wiley & Sons, Ltd.

**Abbreviations used**
ADMA: asymmetric dimethylarginine
l‐NMMA: *N^G^*‐monomethyl‐l‐arginine
MEA: monoethyl‐l‐arginine
OPA: *ortho*‐phthaldialdehyde
PMT: photomultiplier tube
PRMT: protein arginine methyltransferase
RF: response factor
SDMA: symmetric dimethylarginine
SPE: solid‐phase extraction

## Introduction

Protein‐incorporated arginine residues may be methylated post‐translationally by protein arginine methyltransferases (PRMTs; Kakimoto and Akazawa, 1970; Lee *et al*., 1977). Subsequent hydrolytic breakdown of methylated proteins results in the release of three methylarginine derivatives: *N^G^*,*N^G^*‐dimethyl‐l‐arginine (asymmetric dimethylarginine, ADMA), *N^G^*‐monomethyl‐l‐arginine (l‐NMMA) and *N^G^*,*N* ^*G*’^‐dimethyl‐l‐arginine (symmetric dimethylarginine, SDMA). ADMA is metabolized to dimethylamine and citrulline by dimethylarginine dimethylaminohydrolase (DDAH; Achan *et al*., 2003) or cleared by renal excretion (Vallance *et al*., 1992). l‐NMMA and SDMA are also excreted by the kidney, but are not degraded by DDAH (Vallance *et al*., 1992).

Nitric oxide (NO) production by nitric oxide synthase (NOS) requires l‐arginine as a substrate and is inhibited by ADMA and l‐NMMA (Cardounel *et al*., 2007). Furthermore, ADMA, l‐NMMA and SDMA inhibit cellular uptake of arginine, which could limit NOS substrate availability (Closs *et al*., 1997). In humans, ADMA infusion decreases forearm blood flow, decreases plasma cGMP, increases mean arterial blood pressure and increases systemic vascular resistance (Vallance *et al*., 1992; Achan *et al*., 2003; Kielstein *et al*., 2004). Plasma ADMA is inversely correlated with endothelial function in hypercholesterolemia (Böger *et al*., 1998) and hypertension (Perticone *et al*., 2005). Elevated plasma ADMA increases risk of complications and death in sickle‐cell disease (Kato *et al*., 2009), coronary artery disease (Schnabel *et al*., 2005), stroke (Worthmann *et al*., 2011) and end‐stage renal disease (Zoccali *et al*., 2001). SDMA does not inhibit NO synthesis, but inhibits cellular arginine uptake (Closs *et al*., 1997) and serves as a biomarker of renal dysfunction (Kielstein *et al*., 2006; Bode‐Böger *et al*., 2006). To support further investigation of methylarginine accumulation in diseases characterized by endothelial dysfunction, we developed a high‐performance liquid chromatography (HPLC) assay to quantify arginine, ADMA, l‐NMMA and SDMA in a single analytical run.

While several validated HPLC methods have been published (Heresztyn *et al*., 2004; Zhang and Kaye, 2004; Marra *et al*., 2003; Blackwell *et al*., 2009; Teerlink *et al*., 2002; Jones *et al*., 2010), we have addressed five key analytical goals that have not previously been met in a single assay. First, existing assays typically require 100–200 μL of plasma sample for extraction and analysis, a prohibitive requirement for studies utilizing archived plasma samples, mouse models of disease or other limitations on sample volume. Second, we have separated target metabolites from other plasma amino acids. As a result, the accuracy of methylarginine quantification will be preserved if sample processing fails to completely remove acidic or neutral amino acids. Third, we have validated our method for analysis of mouse as well as human plasma, expanding the application of this assay to basic animal model research in addition to clinical studies. Fourth, we utilized the nonendogenous compound monoethyl‐l‐arginine (MEA) as an internal standard following a previously published approach (Blackwell *et al*., 2009). Fifth, we utilized automated derivatization and validated reproducibility of results over a 24 h period, allowing a daily workflow cycle to support continuous throughput of sample analysis.

## Materials and methods

### Ethical approval of human and animal research

Adult subjects provided written informed consent in accordance with the Declaration of Helsinki and were enrolled at the National Institutes of Health Clinical Center on clinical protocol NIH 03‐H‐0015 specifically approved for this study by the Institutional Review Board of the National Heart, Lung and Blood Institute. Blood was drawn into Vacutainers® containing EDTA and then centrifuged at 1500 ***g*** for 5 min to separate plasma from red blood cells. Plasma was stored at −80 °C until analysis.

Animal experiments were performed at the National Institutes of Health (NIAID Comparative Medicine Branch) using a protocol approved by the NIH Animal Care and Use Committee under the designation LMVR 18E. Ten‐week‐old C57BL/6 J male mice were obtained from the Jackson Laboratory (Bar Harbor, ME, USA). Mice were housed in temperature‐controlled cages maintained at 20–22 °C with a 12/12 h light–dark cycle and free access to water and autoclaved rodent feed pellets (Teklad Global 18% Protein Extruded Rodent Diet, 2018SX, Harlan Laboratories, Frederick, MD, USA). Blood was drawn from the inferior vena cava using a syringe containing EDTA. Plasma was separated from erythrocytes by centrifugation at 3000 ***g*** for 5 min at 4 °C before being stored at −80 °C until analysis.

### Instrumentation

All HPLC instrument modules were manufactured by Agilent Technologies (Santa Clara, CA, USA). The instrument consisted of a G1379A microdegasser, a G1376A capillary flow binary pump with a zero‐volume union used to bypass the micro‐flow meter, a G1377 low‐flow, high‐precision autosampler with a G1330B sample plate temperature‐control unit, a G1316A column temperature‐control unit and a G1321B fluorescence detector with a 4 μL flow cell. Agilent Chemstation OpenLab CDS revision C.01.03 was used to control the instrument and acquire data. Analytes were separated on a 1.0 × 100 mm Luna® C_18_(2) column with 3 µm particles and 100 Å pore size (Phenomenex, Torrance, CA, USA). A 1.0 × 13 mm ACE® C_18_ guard column with 3 µm particles and 100 Å pore size (MAC‐MOD Analytical, Chadds Ford, PA, USA) was installed to protect the primary column.

### Reagents

Ultrapure water was obtained from a Milli‐Q Synthesis system (EMD Millipore, Billerica, MA, USA). HPLC‐grade acetonitrile and methanol were obtained from Fisher Scientific (Pittsburgh, PA, USA). For solid‐phase extraction, 0.1 m HCl was prepared in ultrapure water from 37% HCl (Sigma‐Aldrich, St Louis, MO, USA), and elution buffer was prepared from ultrapure water, HPLC‐grade methanol and 28–30% ammonium hydroxide (Sigma‐Aldrich, St. Louis, MO, USA) in a ratio of 5:4:1 H_2_O:MeOH:NH_4_OH. For chromatography, aqueous mobile phase (solvent A) consisted of 25 mm sodium phosphate, pH 6.8 prepared from sodium phosphate dibasic (Acros Organics, Geel, Belgium) and sodium phosphate monobasic (Acros Organics, Geel, Belgium), and 5% v/v HPLC‐grade acetonitrile (Fisher Scientific, Pittsburgh, PA, USA) in ultrapure water. Solvent B was 50% ultrapure water and 50% HPLC‐grade acetonitrile. The derivatization reagent was prepared from *ortho*‐phthaldialdehyde (Sigma‐Aldrich, St Louis, MO, USA) dissolved in HPLC‐grade methanol to a concentration of 50 mg/mL (372.8 mm), 3‐mercaptopropionic acid (Sigma‐Aldrich, St Louis, MO, USA) and 200 mm potassium tetraborate, pH 9.4 (Sigma‐Aldrich, St Louis, MO, USA). A 5‐fold concentrated solution was prepared by adding 50 μL of 50 mg/mL *ortho*‐phthaldialdehyde (OPA) in methanol and 6 μL 3‐mercaptopropionic acid to 444 μL of 200 mm potassium tetraborate. Prior to use in analysis, this 5‐fold concentrated solution was diluted 1/5 in 200 mm potassium tetraborate, yielding a working solution of 7.46 mm OPA and 27.54 mm 3‐mercaptopropionic acid. For make‐up of standards of known concentrations, l‐arginine was from Sigma‐Aldrich (St Louis, MO, USA), l‐NMMA, ADMA and SDMA were from Calbiochem (EMD Millipore, Billerica, MA, USA) and MEA was from Enzo Life Sciences (Farmingdale, NY, USA). l‐arginine, l‐NMMA, ADMA, SDMA and MEA were weighed, dissolved in ultrapure water and stored at −80 °C until use.

### Solid‐phase extraction

Solid‐phase extraction (SPE) was performed with a Waters Oasis MCX 96‐well μElution Plate, 2 mg sorbent per well, 30 µm particle size (Waters Corporation, Milford, MA, USA). Vacuum was applied with an extraction plate manifold (Waters Corporation, Milford, MA, USA). Wells were conditioned with 250 μL elution buffer and equilibrated with 300 μL ultrapure water. Plasma samples were thawed, centrifuged at 16,100 ***g*** for 5 min at 4 °C and 12 μL was added to 42 μL of 1 × phosphate‐buffered saline without Mg^2+^ or Ca^2+^ (Lonza, Walkersville, MD, USA) containing 11.43 µm MEA (for a final concentration of 40 µm after extraction, drying and reconstituting). A 45 μL aliquot was loaded into the SPE well and applied to the sorbent under low vacuum (−50 to −100 mmHg). Each well was subsequently washed with 250 μL 0.1 m HCl and 300 μL methanol. Analytes were then eluted with 250 μL elution buffer and dried at 45 °C under vacuum for 3.5–4 h. Dried samples were then reconstituted in 10 μL ultrapure water and loaded into screw‐capped polypropylene sample vials (Agilent Technologies, Santa Clara, CA, USA) for analysis.

### Deproteination

For chromatographic investigation, some plasma samples were deproteinated by mixing 1:1 with 2 m perchloric acid (HClO_4_, Fisher Scientific, Pittsburgh, PA, USA), centrifuging for 5 min at 16,100 ***g*** to remove protein precipitate and neutralizing with 1.5 m potassium carbonate (Sigma‐Aldrich, St Louis, MO, USA).

### On‐line derivatization

*Ortho*‐phthaldialdehyde derivatization is a common method used to facilitate chromatographic separation and fluorescent detection of metabolites with primary amines, including amino acids (Molnar‐Perl, 2001). In our assay, the autosampler was programmed to draw 1.7 μL of OPA and 3‐mercaptopropionic acid derivatization solution and 1.7 μL of the current sample in the sequence, mix the contents in the needle for 30 s and inject the derivatized sample onto the column. Before and after each of these steps, the needle was washed for 5 s with 10% HPLC‐grade methanol diluted in ultrapure water using the built‐in autosampler flushport to prevent carry‐over contamination.

### Chromatography and fluorescence detection

Chromatographic separation was performed isocratically for 20 min followed by a gradient from 0% B at 20 min to 18% B at 42 min. The column was then washed with 100% solvent B for 5 min and re‐equilibrated with 0% B for 13 min prior to injection of the following sample. The column was maintained at 35 °C for all runs. Online fluorescence was measured at excitation and emission wavelengths of 340 and 455 nm, respectively. The fluorescence detector's photomultiplier tube (PMT) gain was set to 11 for initial normal‐sensitivity analysis. At 20 min, PMT gain was switched to 16 for the detection of low‐concentration analytes (MMA, ADMA and SDMA). At 37 min PMT gain was switched back to 11 for detection of MEA with normal sensitivity. Chemstation OpenLab CDS software version C.01.03 was used for peak integration.

### Quantification of sample analytes

Internal and external standards were used for quantification of analytes. A combined standard of 50 µm arginine, 0.5 µm l‐NMMA, 0.5 µm ADMA and 0.5 µm SDMA was spiked with the internal standard MEA, extracted, dried, reconstituted and analyzed in parallel with each set of samples. Based on the peak areas of this combined standard, a response factor (RF) was calculated for each amino acid in the external standard:

(1) $$RF=\frac{\text{analyte peak are}a_{\text{external}\;std}}{\left[\text{external}\;std\right]}$$

where [external std] is 50 µm for arginine and 0.5 µm for l‐NMMA, ADMA and SDMA.

Monoethylarginine (40 µm) was used as an internal standard. Concentrations of amino acid analytes in the sample were calculated from the external standard response factor and normalized to recovery of MEA:

(2) $${\left[\text{Analyte}\right]}_{\text{sample}}=\frac{\text{analyte peak are}a_{\text{sample}}}{RF}\times \frac{MEA\;\text{peak are}a_{\text{standard}}}{MEA\;{\text{peak area}}_{\text{sample}}}$$

### Precision

Precision was calculated from five parallel analyses of human and mouse plasma samples analyzed on the same (intra‐day precision) or different days (inter‐day precision). The coefficient of variation (CV) was calculated and expressed as a percentage:

(3) $$CV=\frac{s}{\bar{x}}\times 100\%$$

where *s* is the standard deviation and *x̄* is the mean of repeated measurements.

### Limit of detection

The limit of detection (LOD) describes the lowest concentration that can be distinguished from baseline noise with a degree of confidence, generally set at 95%. The LOD was determined by assaying *n* = 3 independently prepared water blank samples and *n* = 3 independently prepared combined standards containing 1.0 µm arginine, 0.01 µm l‐NMMA, 0.01 µm ADMA and 0.01 µm SDMA. The limit of detection was calculated according to the following equation (Armbruster and Pry, 2008):

(4) $$LOD=mean_{\text{blank}}+1.645\times SD_{\text{blank}}+1.645\times SD_{\text{low standard}}$$

### Spike Recovery

Recovery of exogenous standards from spiked samples was calculated as an absolute concentration and as a percentage of the known concentration of the standard:

(5) $$\text{Recovery}=\left(\frac{{\left[\text{analyte}\right]}_{\text{sample}+\text{spike}}-{\left[\text{analyte}\right]}_{\text{sample alone}}}{\left[\text{spike}\right]}\right)\times 100\%$$

## Results

### Solid‐phase extraction recovery

ADMA, l‐NMMA and SDMA were present in submicromolar concentrations in the plasma of healthy individuals. To assess the recovery of our sample preparation protocol, we analyzed standards prepared with or without SPE. Absolute recoveries for all metabolites (including MEA) ranged from 95.8% to 103.0% and relative recoveries (normalized to MEA) ranged from 93.9% to 101.0% (Table S1 in the Supporting Information). These results indicated sufficient recovery to proceed with assay development.

### Chromatography

**Figure 1:**
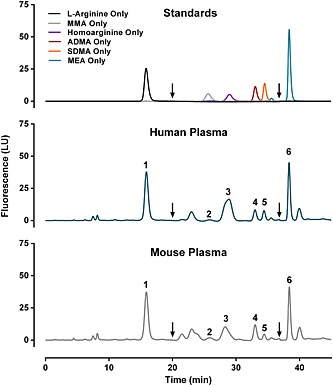
Resolution and identification of analytes. Arginine (50 µm),*N^G^*‐monomethyl‐l‐arginine (l‐NMMA; 0.5 µm), homoarginine (0.5 µm), asymmetric dimethylarginine (ADMA; 0.5 µm), symmetric dimethylarginine (SDMA; 0.5 µm) and monoethyl‐l‐arginine (MEA; 50 µm) were analyzed individually to determine retention times. Peak identities: 1, arginine; 2, l‐NMMA; 3, homoarginine; 4, ADMA; 5, SDMA; and 6, monoethyl‐Larginine (MEA, internal standard). Concentrations in human plasma (middle panel) were 89.7 µm arginine, 0.09 µm l‐NMMA, 0.35 µm ADMA and 0.28 µm SDMA. Concentrations in mouse plasma (bottom panel) were 83.7 µm arginine, 0.14 µm l‐NMMA, 0.51 µm ADMA and 0.13 µm SDMA. Arrows at 20 and 37 min indicate switch to increased sensitivity (PMT gain 16) and back to normal sensitivity (PMT gain 11), respectively.

Peak identities and retention times were determined by separate analysis of individual arginine, l‐NMMA, homoarginine, ADMA, SDMA and MEA standards (Fig. 1). Chromatographic conditions were designed to achieve baseline separation of arginine, l‐NMMA, ADMA, SDMA and MEA (internal standard) in both human and mouse plasma samples (Fig. 1). The homoarginine peak exhibited evidence of minor contamination in the form of irregular and asymmetric peak shape in human and mouse plasma samples (Fig. 1). For this reason, homoarginine was excluded from further consideration in assay validation.

**Figure 2:**
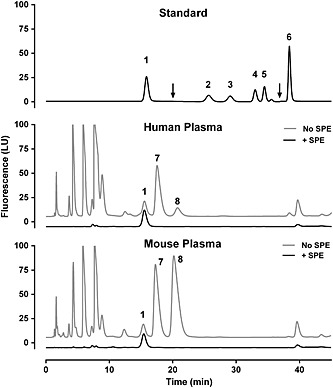
Survey of human and mouse plasma for potential contaminants. Human and mouse plasma were processed with protein precipitation [no solid‐phase extraction (SPE)] or with solid phase extraction using mixed‐mode cation exchange columns (+SPE). For these analyses, plasma samples were not spiked with MEA (internal standard) and photomultiplier tube (PMT) gain remained constant (11) for the duration of the run. Plasma samples were compared with a combined standard (top panel) to identify potential contaminants that may co‐elute with target analytes if not fully eliminated by SPE. Arrows at 20 and 37 min indicate switch to increased sensitivity (PMT gain 16) and back to normal sensitivity (PMT gain 11), respectively. Sensitivity was not increased in the bottom two panels and traces were artificially shifted up (no SPE) and down (+SPE) to aid visualization. Peak identities: 1, arginine; 2, l‐NMMA; 3, homoarginine; 4, ADMA; 5, SDMA; 6, MEA; 7, alanine; and 8, taurine.

We screened for high‐concentration plasma metabolites that contain primary amines that react with the OPA derivatization reagent. Without effective separation, even small amounts of such contaminants inadvertently retained during solid phase extraction could significantly distort quantification of submicromolar methylarginine concentrations in human and mouse plasma. To screen for such compounds, we processed human and mouse plasma samples in parallel by (a) SPE or (b) perchloric acid acidification and potassium carbonate neutralization to deproteinate samples for full amino acid analysis. Using this approach, we identified alanine and taurine as two high‐concentration amine‐containing compounds present in human and mouse plasma (Fig. 2). The chromatographic conditions of our assay ensure that both alanine and taurine were effectively separated from all target metabolites (Fig. 2).

We also observed that the SPE resin contributed a contaminant of unknown identity whether a standard, plasma or water sample was extracted by SPE (Fig. S1 in the Supporting Information). We optimized the chromatographic conditions to ensure separation of l‐NMMA from this unknown SPE contaminant (Fig. S1).

### Standard curve linearity and LOD

**Figure 3:**
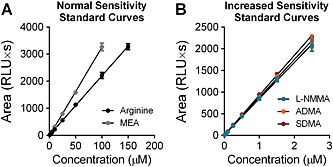
Plot of standard curves. 9‐point dilutions of a combined external standard were prepared and assayed in triplicate. Combined standards contained arginine, l‐NMMA, ADMA, SDMA and MEA (internal standard). The fluorescence detector photomultiplier tube gain was set to 11 for peaks detected at normal sensitivity curves (A) and to 16 for peaks detected with increased sensitivity (B). In each dilution, arginine and MEA were 100× more concentrated than l‐NMMA, ADMA and SDMA. *R* ^2^ values were 0.9998 for arginine, 0.9998 for MEA, 0.9995 for l‐NMMA, 0.9996 for ADMA and 0.9995 for SDMA.

To assess linearity, a combined standard solution containing arginine, l‐NMMA, ADMA, SDMA and MEA was serially diluted to yield a nine‐point standard curve. At each point, l‐NMMA, ADMA and SDMA were one‐hundredth of the concentration of arginine and MEA, consistent with the difference in order of magnitudes observed in plasma. Standard curves for all analytes were highly linear with *R* ^2^ values of >0.9995 for all metabolites (Fig. 3). With the selected chromatographic and fluorescent detection conditions, arginine was off‐scale above 150 µm and MEA was off‐scale above 100 µm. The limit of detection was 0.21 µm for arginine, 0.025 µm for l‐NMMA, 0.007 µm for ADMA and 0.005 µm for SDMA.

### Intra‐ and inter‐day precision

Intra‐day CV values were <5% for all analytes in both human and mouse plasma (Table S2). Inter‐day CV values were ≤5% for arginine, ADMA and SDMA in human plasma and for arginine and ADMA in mouse plasma (Table S2). The inter‐day CV values for SDMA and l‐NMMA in mouse plasma were slightly higher (5.7% and 15.8%, respectively) because their mean concentrations were substantially lower than ADMA (0.12 µmol/L for SDMA and 0.15 µmol/L for l‐NMMA vs 0.53 µmol/L for ADMA, Table S2). Similarly, the inter‐day CV of l‐NMMA was 10.4% in human plasma in association with a mean concentration of 0.17 µmol/L (compared with 0.50 µmol/L for ADMA, Table S2).

### Stability of dissolved analytes

We employed automated derivatization of samples directly prior to injection onto the column. This approach is preferable to manual batch derivatization (Teerlink *et al*., 2002) because it provides a consistent, fixed time interval between derivatization and analysis. This approach avoids the possibility that different decay rates among batch‐derivatized target analytes and internal standards could return inconsistent results in later samples.

We analyzed the stability of dissolved analytes maintained at 4 °C in the autosampler over a 24 h period by comparing repeat analyses of *n* = 3 combined amino acid standards at 0, 12 and 24 h intervals. At the 24 h time point, measurements of all analytes were >97% of initial (0 h), demonstrating that analytes and OPA derivatization solution are stable at 4 °C for 24 h (Table S3).

### Sample matrix effects

Sample matrix effects were assessed by spiking known concentrations of standards into human and plasma mouse samples. Recovery values are tabulated for human plasma samples in Table S4 and for mouse plasma samples in Table S5. Across two concentrations of spiked standards, recovery was within ±5% for arginine, l‐NMMA, ADMA and SDMA in human plasma (Table S4) and for ADMA in mouse plasma (Table S5). Recoveries were within ±10% for arginine, l‐NMMA and SDMA in mouse plasma (Table S5). The standard deviation of l‐NMMA recovery in mouse plasma expressed as a percentage was relatively high for samples spiked with 0.5 µmol/L exogenous standard (±15.2%) owing to low peak areas associated with low endogenous concentration. The standard deviation of l‐NMMA recovery improved to ±4.7% in mouse plasma spiked with 1.0 µmol/L exogenous standard.

## Discussion

We have described an HPLC method capable of quantifying arginine, ADMA, l‐NMMA and SDMA in a single analytical run. ADMA accumulation has been identified as a biomarker and potential mediator of endothelial dysfunction and cardiovascular disease (Zoccali *et al*., 2001; Schnabel *et al*., 2005; Kato *et al*., 2009; Worthmann *et al*., 2011), leading to the suggestion that routine measurement of ADMA could provide clinically useful information (Kielstein and Cooke, 2007). As a competitive inhibitor, the effect of ADMA accumulation on NOS activity is also determined by relative arginine concentrations (Cardounel *et al*., 2007). It is therefore necessary to simultaneously quantify arginine and ADMA, which cannot be accomplished by ELISA‐based quantification of ADMA (Schulze *et al*., 2004).

ADMA, l‐NMMA and SDMA are present in human and mouse plasma in submicromolar concentrations. We addressed this challenge by using mixed‐mode cation exchange SPE to isolate basic plasma metabolites, including ADMA, l‐NMMA and SDMA. Using this approach, we achieved >95% and >93% absolute and relative recoveries, respectively, indicating near‐complete retention and elution of target metabolites during SPE. Previous published methods reported absolute recoveries in the range of 85% using SPE columns from the same manufacturer and similar loading, washing and elution conditions (Teerlink *et al*., 2002). We speculate that we were able to achieve improved recoveries primarily by applying low vacuum during sample loading and washing. This approach increases the duration of column saturation by samples and solvents, ensuring that binding and elution reactions proceed to completion. Our method also achieved improved lower limit of detection and standard curve linearity.

The primary advantage of our method is the use of minimal sample volume (12 μL). Published methods require sample volumes ranging from 100 μL (Jones *et al*., 2010) to 200 μL (Teerlink *et al*., 2002; Marra *et al*., 2003; Blackwell *et al*., 2009; Suzuki *et al*., 2013), and in some cases 500 μL (Heresztyn *et al*., 2004). Such sample requirements can be prohibitive for research applications where sample volume is limited, such as analysis of archived clinical samples or plasma from mouse model studies. Through venipuncture of the inferior vena cava under terminal anesthesia, we routinely obtain 700‐800 μL of whole blood from 10‐week‐old male C57BL/6 mice (~25 g), yielding approximately 300–350 μL of plasma that may be obtained without disturbing the buffy coat. The minimal volume requirements of our assay provide the opportunity to conduct additional biochemical analyses that may be limited by assays with higher volume requirements.

While use of a nonendogenous standard is best practice for quantitative biochemical analysis, endogenous metabolites such as l‐NMMA have been used in previous HPLC‐based methodologies (Teerlink *et al*., 2002). The use of l‐NMMA as an internal standard precludes quantification of endogenous l‐NMMA concentrations. In addition, physiological or pathophysiological variation in plasma l‐NMMA concentrations would produce error in arginine, ADMA and SDMA values normalized to the l‐NMMA internal standard, although this error could be minimized by addition of high l‐NMMA concentrations. To avoid these drawbacks, we utilized the nonendogenous arginine derivative MEA as an internal standard, following the approach of Blackwell *et al*. (2009).

The primary disadvantages of our method are the significant sample processing requirements and relatively long chromatographic run time (55 min). The extended run time was required to effectively separate l‐NMMA from the unknown SPE contaminant and from arginine and taurine, which may not be completely removed during sample clean‐up. Use of low‐flow conditions also required a relatively long re‐equilibration period (13 min). To accommodate the relatively long run time, we verified the stability of target metabolites and the OPA/3‐mercaptopropionic derivatization reagent over 24 h. This data supports use of a 24 h work‐flow where samples may be processed daily and analyzed overnight.

A number of mass spectrometry‐based assays for methylarginine derivatives have been published in recent years (Kirchherr and Kühn‐Velten, 2005; Martens‐Lobenhoffer and Bode‐Böger, 2003; Schwedhelm *et al*., 2007; Bishop *et al*., 2007; El‐Khoury *et al*., 2012; Gervasoni *et al*., 2011; Servillo *et al*., 2013). Compared with HPLC‐based methods, mass spectrometry‐based analysis typically requires less intensive sample processing and allows for shorter run times. Despite these advantages, mass spectrometry requires greater investment in instrumentation and maintenance, highlighting the importance of guidelines for thorough HPLC method development and validation, as we have described here. Alternatively, electrochemical detection has been utilized to detect arginine and methylarginines (Suzuki *et al*., 2013). According to this report, electrochemical detection improves the limit of detection to 0.5 pmol (vs 0.025 µm for l‐NMMA, 0.007 µm for ADMA and 0.005 µm for SDMA in our assay). However, the chromatography conditions utilized in this assay (Wako Combi ODS column and 1:1, v/v acetonitrile–100 mm sodium phosphate buffer, pH 7.0) were unable to produce baseline separation between l‐NMMA and arginine (Suzuki *et al*., 2013). We suggest that adaptation of our running conditions could improve the performance of this assay, especially with regard to l‐NMMA quantification.

In summary, we have developed and validated a chromatographic method for extracting, separating and quantifying arginine, l‐NMMA, ADMA and SDMA with the unique advantages of small sample volume requirements, excellent separation of target metabolites from potential contaminants and validation for both human and mouse plasma samples.

## Author contributions

M.S.A., G.N. and H.C.A. conceived the analytical approach; M.S.A. and G.N. developed the assay; M.S.A. validated the assay; J.H.C. assisted with sample preparation

## Supporting information



Supporting info item
