# Supplementary material for: Resolution and quantification of arginine, monomethylarginine, asymmetric dimethylarginine, and symmetric dimethylarginine in plasma using HPLC with internal calibration
Source: Biomed Chromatogr. 2015 Jul 30;30(3):294–300. doi: 10.1002/bmc.3548 (PMC4755038; doi:10.1002/bmc.3548)
Supplement: Supplementary file 1 — Supporting info item [file BMC-30-294-s001.docx]

**Supplementary Figures**

**
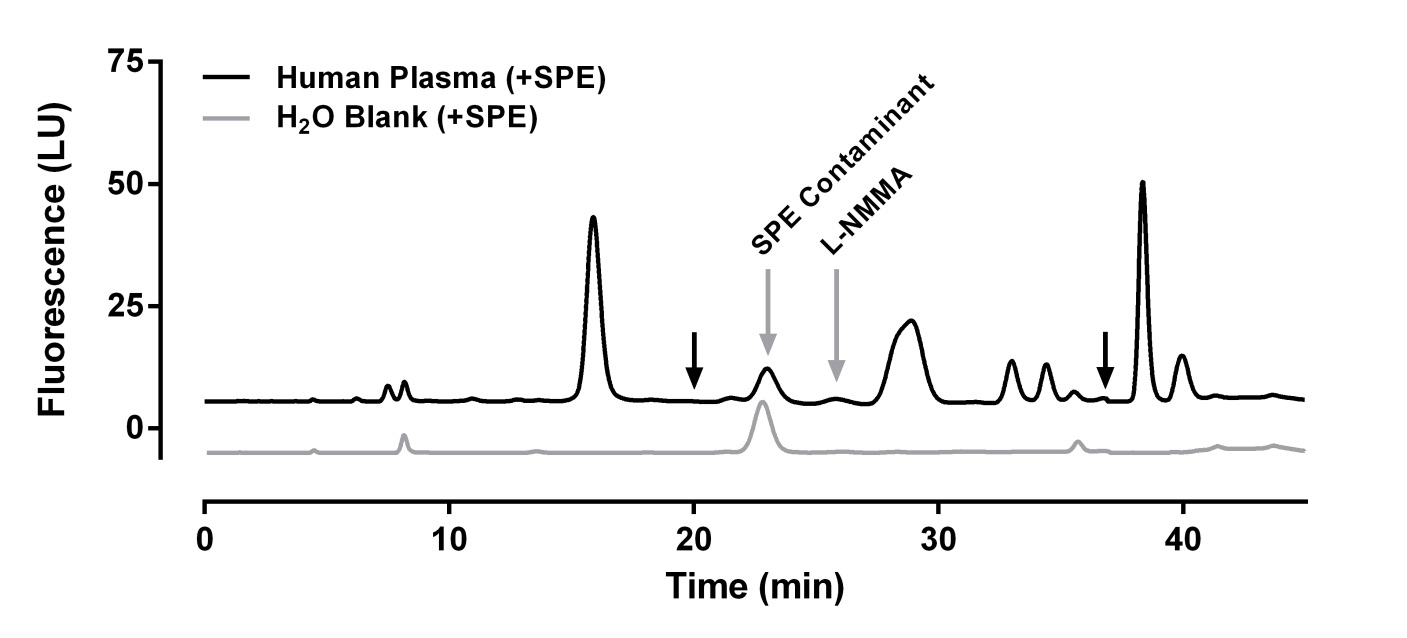
**

**Figure S1. Chromatographic separation of contaminant contributed by SPE column.** Under the selected chromatographic conditions (isocratic separation with 5% acetonitrile for first 20min), an unknown contaminant contributed by the solid phase extraction column was well-separated from L-NMMA. The H_2_O blank was generated by performing solid phase extraction on an aliquot of ultrapure water. Traces are artificially shifted up (human plasma) or down (H_2_O blank) to aid visualization.

**Supplementary Tables**

|  |  | **Absolute Recovery (%)** | | |  | **Relative Recovery (%)** | | |
| --- | --- | --- | --- | --- | --- | --- | --- | --- |
| **Analyte** |  |  |  |  |  |  |  |  |
| Arg |  | 98.3 | ± | 2.8 |  | 96.4 | ± | 2.1 |
| L-NMMA |  | 95.8 | ± | 2.8 |  | 93.9 | ± | 1.7 |
| ADMA |  | 103.0 | ± | 2.5 |  | 101.0 | ± | 1.9 |
| SDMA |  | 100.8 | ± | 3.3 |  | 98.8 | ± | 2.6 |
| MEA |  | 102.0 | ± | 2.0 |  | 100.0 |  |  |

**Table S****1. Absolute and relative recovery of analytes from solid phase extraction.** Standards (n=12) containing 50μM arginine, 0.5μM L-NMMA, 0.5μM ADMA, 0.5μM SDMA and 50μM MEA were extracted with strong cation exchange column. Absolute recovery values reflect integrated areas expressed as a percentage of mean integrated analyte peak areas ± SD from n=5 repeat analyses of the original standard. Relative recovery values are normalized to the internal standard MEA.

| **Intra-day Precision** | | | | | | | | | | | | | |
| --- | --- | --- | --- | --- | --- | --- | --- | --- | --- | --- | --- | --- | --- |
|  |  | **Human plasma** | | | | | |  | **Mouse plasma** | | | | |
| **Analyte** |  | **Concentration (μmol/L)** | | | |  | **%CV** |  | **Concentration (μmol/L)** | | |  | **%CV** |
| Arg |  | 90.9 | | ± | 1.8 |  | 1.9 |  | 90.3 | ± | 1.3 |  | 1.4 |
| L-NMMA |  | 0.13 | | ± | 0.00 |  | 2.0 |  | 0.16 | ± | 0.01 |  | 4.0 |
| ADMA |  | 0.42 | | ± | 0.01 |  | 2.2 |  | 0.53 | ± | 0.01 |  | 1.9 |
| SDMA |  | 0.41 | | ± | 0.01 |  | 2.9 |  | 0.12 | ± | 0.00 |  | 2.4 |
|  |  |  | |  |  |  |  |  |  |  |  |  |  |
| **Inter-day Precision** | | | | | | | | | | | | | |
|  |  | **Human plasma** | | | | | |  | **Mouse plasma** | | | | |
| **Analyte** |  | **Concentration (μmol/L)** | | | |  | **%CV** |  | **Concentration (μmol/L)** | | |  | **%CV** |
| Arg |  | 98.0 | ± | | 4.4 |  | 4.5 |  | 87.9 | ± | 2.9 |  | 3.3 |
| L-NMMA |  | 0.17 | ± | | 0.02 |  | 10.4 |  | 0.15 | ± | 0.02 |  | 15.8 |
| ADMA |  | 0.50 | ± | | 0.02 |  | 3.8 |  | 0.53 | ± | 0.02 |  | 3.1 |
| SDMA |  | 0.47 | ± | | 0.02 |  | 5.0 |  | 0.12 | ± | 0.01 |  | 5.7 |

**Table S****2. Intra- and inter-day precision.** Human mouse and plasma samples were repeatedly analyzed 5 times on the same day (intra-day measurements) on 5 different days (inter-day measurements). Measured concentrations are reported as mean ± SD for repeated analyses and precision was assessed by calculating the coefficient of variation as a percent (%CV).

| **Analyte stability (% of Initial at 0hr)** | | | | | | | | |
| --- | --- | --- | --- | --- | --- | --- | --- | --- |
| **Analyte** |  | **12hr** | | |  | **24hr** | | |
| Arg |  | 99.83 | ± | 0.66 |  | 99.66 | ± | 1.11 |
| L-NMMA |  | 100.08 | ± | 0.72 |  | 99.33 | ± | 0.69 |
| ADMA |  | 99.41 | ± | 0.30 |  | 98.58 | ± | 0.11 |
| SDMA |  | 99.04 | ± | 0.18 |  | 97.89 | ± | 0.49 |
| MEA |  | 100.11 | ± | 0.52 |  | 100.05 | ± | 0.72 |

**Table S****3. Analyte stability at 4°C in the autosampler.** Values are expressed as mean % of initial at 0hr ±SD. Data were determined from repeat assessments of three independent standards consisting of 50μM arginine, 0.5μM L-NMMA, 0.5μM ADMA, 0.5μM SDMA and 50μM MEA.

| **Human Plasma** | | | | | | | | | | | | | |
| --- | --- | --- | --- | --- | --- | --- | --- | --- | --- | --- | --- | --- | --- |
|  | **Spike Added (μM)** |  | **Measured Concentration (μM)** | | |  | **Recovery** | | | | | | |
|  |  |  |  |  |  |  | **Concentration (μM)** | | |  | **Percent of initial (%)** | | |
| **Analyte** |  |  |  |  |  |  |  |  |  |  |  |  |  |
| **Arginine** | - |  | 90.0 | ± | 1.1 |  | - |  |  |  | - |  |  |
|  | 50.0 |  | 141.2 | ± | 2.5 |  | 51.1 | ± | 2.5 |  | 102.3 | ± | 5.0 |
|  | 100.0 |  | 186.8 | ± | 3.8 |  | 96.8 | ± | 3.8 |  | 96.8 | ± | 3.8 |
|  |  |  |  |  |  |  |  |  |  |  |  |  |  |
| **L-NMMA** | - |  | 0.13 | ± | 0.00 |  |  |  |  |  |  |  |  |
|  | 0.5 |  | 0.62 | ± | 0.00 |  | 0.49 | ± | 0.00 |  | 97.1 | ± | 0.8 |
|  | 1.0 |  | 1.09 | ± | 0.02 |  | 0.96 | ± | 0.02 |  | 95.7 | ± | 2.1 |
|  |  |  |  |  |  |  |  |  |  |  |  |  |  |
| **ADMA** | - |  | 0.43 | ± | 0.01 |  |  |  |  |  |  |  |  |
|  | 0.5 |  | 0.92 | ± | 0.01 |  | 0.50 | ± | 0.01 |  | 99.4 | ± | 1.3 |
|  | 1.0 |  | 1.38 | ± | 0.02 |  | 0.95 | ± | 0.02 |  | 95.4 | ± | 2.3 |
|  |  |  |  |  |  |  |  |  |  |  |  |  |  |
| **SDMA** | - |  | 0.40 | ± | 0.01 |  |  |  |  |  |  |  |  |
|  | 0.5 |  | 0.92 | ± | 0.01 |  | 0.52 | ± | 0.01 |  | 104.5 | ± | 1.4 |
|  | 1.0 |  | 1.41 | ± | 0.02 |  | 1.01 | ± | 0.02 |  | 101.4 | ± | 2.1 |

**Table S****4. Recovery of amino acid standards added to human plasma.** Recovery was calculated by quantifying plasma samples with or without addition of amino acid standards at the indicated concentrations. Spikes were performed in three aliquots of the same plasma samples per condition. Recovery was calculated as a percentage of the known concentration of the added standards. All values are mean ± SD.

| **Mouse Plasma** | | | | | | | | | | | | | |
| --- | --- | --- | --- | --- | --- | --- | --- | --- | --- | --- | --- | --- | --- |
|  | **Spike Added (μM)** |  | **Measured Concentration (μM)** | | |  | **Recovery** | | | | | | |
|  |  |  |  |  |  |  | **Concentration (μM)** | | |  | **Percent of initial (%)** | | |
| **Analyte** |  |  |  |  |  |  |  |  |  |  |  |  |  |
| **Arginine** | - |  | 87.7 | ± | 3.0 |  | - |  |  |  | - |  |  |
|  | 50.0 |  | 140.8 | ± | 3.0 |  | 53.1 | ± | 3.0 |  | 106.3 | ± | 6.0 |
|  | 100.0 |  | 189.0 | ± | 9.4 |  | 101.3 | ± | 9.4 |  | 101.3 | ± | 9.4 |
|  |  |  |  |  |  |  |  |  |  |  |  |  |  |
| **L-NMMA** | - |  | 0.16 | ± | 0.00 |  |  |  |  |  |  |  |  |
|  | 0.5 |  | 0.61 | ± | 0.08 |  | 0.45 | ± | 0.08 |  | 90.7 | ± | 15.2 |
|  | 1.0 |  | 1.09 | ± | 0.05 |  | 0.93 | ± | 0.05 |  | 93.5 | ± | 4.7 |
|  |  |  |  |  |  |  |  |  |  |  |  |  |  |
| **ADMA** | - |  | 0.54 | ± | 0.01 |  |  |  |  |  |  |  |  |
|  | 0.5 |  | 1.06 | ± | 0.02 |  | 0.51 | ± | 0.02 |  | 102.9 | ± | 3.2 |
|  | 1.0 |  | 1.56 | ± | 0.06 |  | 1.01 | ± | 0.06 |  | 101.4 | ± | 6.1 |
|  |  |  |  |  |  |  |  |  |  |  |  |  |  |
| **SDMA** | - |  | 0.13 | ± | 0.01 |  |  |  |  |  |  |  |  |
|  | 0.5 |  | 0.68 | ± | 0.03 |  | 0.55 | ± | 0.03 |  | 109.2 | ± | 5.1 |
|  | 1.0 |  | 1.18 | ± | 0.07 |  | 1.05 | ± | 0.07 |  | 104.6 | ± | 7.2 |

**Table S****5. Recovery of amino acid standards added to mouse plasma.** Recovery was calculated by quantifying plasma samples with or without addition of amino acid standards at the indicated concentrations. Spikes were performed in three aliquots of the same plasma samples per condition. Recovery was calculated as a percentage of the known concentration of the added standards. All values are mean ± SD.
